# Molecular Pathology of Pancreatic Cancer

**DOI:** 10.3390/cancers14061523

**Published:** 2022-03-16

**Authors:** Eva Karamitopoulou

**Affiliations:** 1 Pancreatic Cancer Research, Institute of Pathology, University of Bern, CH-3008 Bern, Switzerland; eva.diamantis@patho.ch or eva.diamantis@pathology.unibe.ch; 2 Pancreatobiliary and GI-Pathology, Pathology Institute Enge, CH-8005 Zurich, Switzerland

Pancreatic ductal adenocarcinoma (PDAC) is a biologically aggressive malignancy showing a remarkable resistance to existing therapies and is often diagnosed at an advanced stage, leaving only about 15–20% of patients with an option for surgical resection [1]. Despite major improvements in surgical techniques and chemotherapy regimens, the overall 5-year survival for PDAC is currently <10% [1]. Moreover, its incidence is increasing by up to 1% per year, and it is projected to become the second-leading cause of cancer-related mortality by 2030 [2].

The mutational landscape of PDAC is dominated by recurrent driver mutations in *KRAS*, *TP53*, *SMAD4*, and *CDKN2A*, which occur alone or in combination in >50% of the cases, whereas recurrent mutations in a number of other genes, including *ARID1A*, *KDM6A*, *MLL3*, *TGFBR2*, *RBM10*, and *BCORL1*, are found in <10% of tumors [3,4,5]. Gene alterations associated with targeted therapy such as *ERBB2* amplification; *BRAF* gene fusions/mutations; and mutations in the DNA damage repair (DDR) genes *BRCA1*, *BRCA2*, or *PALB2* are found in a small percentage of PDAC patients [6,7]. Nevertheless, a number of recently identified “actionable molecular phenotypes” is currently under clinical investigation [5]. For example, Casolino and co-workers performing a meta-analysis of 21,842 PDAC genomes has estimated that the pooled prevalence of germline and somatic mutations in DDR genes (i.e., *BRCA1*, *BRCA2*, *PALB2*, *ATM*, *ATR*, *CHEK2*, *RAD51*, and *FANC*) that cause homologous recombination deficiency (HRD) lies between 14.5 and 16.5% of PDAC cases [8]. Moreover, recent evidence suggests that unstable genomes as determined by Structural Variation analysis and *BRCA* mutational signatures (BRCAness phenotype) can also act as surrogate biomarkers of HRD [3,8]. Thus targeting HRD may cover not only the germline carriers but also PDAC patient subsets harboring somatic mutations in DDR genes or even patients exhibiting a “BRCAness phenotype” [8]. Since HRD is a predictive biomarker of response to DNA damaging agents such as platinum and PARP inhibitors, all of the above suggests that up to 44% of PDAC patients might benefit from these therapeutic approaches [5,8]. 

Additionally, “bulk” transcriptomic profiling has identified two broad PDAC subtypes with distinct biology, namely Classical and Basal-like, with Basal-like tumors associated with significantly poorer outcome [4,9,10,11,12,13,14]. These subtypes are characterized by the differential expression of pancreatic specific transcription factors, such as GATA6, PDX1, and HNF1A, which are maintained in Classical tumors and are lost in Basal-like PDACs [4,9,10,11]. Furthermore, these data are expanded by the results of next-generation single cell sequencing (scRNAseq) and single nucleus sequencing (snRNAseq), which provide a comprehensive map of tumor cell subsets and can give us insight into chemotherapy resistance and metastasis [15].

In addition, the tumor microenvironment (TME) of PDAC has been established as an important player affecting disease progression and response to therapy [16,17,18]. The PDAC TME is generally considered “immunologically cold”, exhibiting low numbers of CD8^+^ cytotoxic T cells and high numbers of immunosuppressive immune cell populations, rendering most PDAC patients poor candidates for immunotherapy [19,20]. Indeed, immunotherapy response rates are very low in PDAC, limited in a rare subset of patients with microsatellite instability–high (MSI-high)/mismatch repair–deficient (dMMR) tumors [21,22]. However, new methods such as spatially resolved transcriptomics and multiplexed imaging modalities provide us with substantial information concerning the interactions between tumor and immune cells, revolutionizing our knowledge about the immune microenvironment of PDAC [15]. Moreover, the deconvolution of bulk RNA data using validated gene signatures has demonstrated that many immune cell populations, including T cells, B cells, and myeloid cells, as well as their subtypes contribute to complex and heterogeneous immune profiles in the PDAC TME. Immunophenotyping of PDAC tissues using scRNAseq, spatial transcriptomics, and multiplexed immunofluorescence has revealed that Classical and Basal-like cell phenotypes are associated with distinct immune microenvironments [15]. Thus, Basal-like tumors are associated with increased macrophage infiltration and loss of cytotoxic T cells in both primary and metastatic micro-niches [23] (Figure 1). These findings suggest that Basal-like tumors may respond to therapies that specifically target tumor-associated macrophages (TAMs), such as Colony stimulating factor 1 receptor (CSF1R) inhibitors [24]. However, the propencity of DDR and MSI to induce distinct immune profiles independent of a certain molecular PDAC subtype is currently unknown. Tumor-infiltrating T cells are associated with increased overall survival in PDAC and can potentially predict immunotherapy response [25,26]. Single cell analysis has even showed that CD8^+^ T cell tumor infiltration is inversely correlated with myeloid cell enrichment [17]. However, tumor-infiltrating CD8^+^ T cells can exhibit exhausted phenotypes which can increase with disease progression [26]. Exhausted CD8^+^ T cell signatures were associated with increased expression of the immune checkpoint TIGIT (i.e., T cell immunoglobulin and ITIM domain) [15]. The ligand for TIGIT, PVR (i.e., poliovirus receptor), was expressed in tumor, endocrine, and endothelial cells and myeloid subsets, supporting the observation that myeloid cells promote immunosuppression in PDAC [27]. Recent data also show that immune checkpoint receptors PD-1/PD-L1 (i.e., programmed cell death 1/programmed cell death 1 ligand 1) are heterogeneously expressed in PDAC patients and associated with distinct immune microenvironments [17,26]. In addition, it has been shown that primary PDACs and metastatic lesions have distinct immune microenvironments [23,24,28]. These data highlight the complexity of individual patient immune microenvironments and suggest that therapeutic approaches targeting immune checkpoints may need to be tailored to individual PDAC patients [15,17]. It is also currently unclear how immune microenvironments change during patient treatment. Therefore, longitudinal single-cell studies mapping the variability of the immune microenvironment and the cell–cell interactions between neoplastic and immune cells will be very helpful for the improvement of immunotherapies for PDAC patients.

Many studies have demonstrated that genetic changes, such as *KRAS* and *MYC*, can also modulate the PDAC TME and enhance its immunosuppressive nature [29,30,31]. Changes associated with response to immune checkpoint inhibitors (ICI) such as microsatellite instability have very low prevalence in PDAC (around 1%) [22]. While *BRCA1*- and *BRCA2*-deficient tumors are associated with increased immune infiltrates, the rates of response to ICI are low. Recent evidence in mouse models of breast and colorectal cancer suggest that *BRCA2*-deficient tumors are more susceptible to ICIs than *BRCA1*-deficient tumors [32,33]. In addition, a loss of *CDKN2A*, which is a feature of PDAC, has been identified as a biomarker of immune checkpoint therapy resistance in solid tumors [34]. These studies show that a diversity of events may affect the response to ICIs and suggest that, for the administration of immunotherapy, the complex genomic and biomarker signature of each individual tumor should be taken into consideration.

In conclusion, for the implementation of precision oncology in the management of PDAC patients, the use of appropriate biomarkers in routine clinical care is necessary. Cancer biomarkers detected in tumor tissue, blood, or other fluids can aid in the early detection of PDAC or its recurrence and may have prognostic as well as predictive roles. These biomarkers are still being discovered.

## Figures and Tables

**Figure 1:**
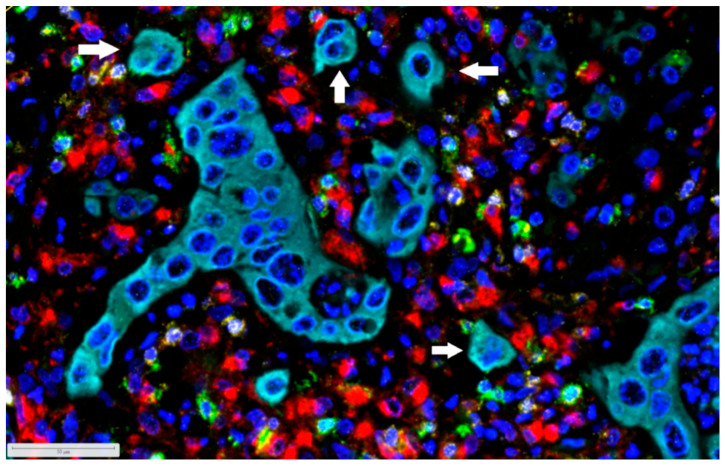
PDAC (pancreatic ductal adenocarcinoma) with Basal-like phenotype with presence of many tumor buds (arrows) and an immunosuppressive microenvironment with many CD68^+^ tumor-associated macrophages (TAMs) and few CD8^+^ and CD3^+^ T cells, many of which express FOXP3 (T regulatory cells; Tregs). Tumor cells (pancytokeratin): cyan; CD68^+^ TAMs: red; CD8^+^ T cells: green; CD3^+^ T cells: yellow; and FOXP3^+^ Tregs: white. DAPI (4′,6-Diamidino-2-phenylindol): blue. Multiplex immunofluorescence ×300.
